# Afferent arteriole responsiveness to endothelin receptor activation: does sex matter?

**DOI:** 10.1186/s13293-018-0218-2

**Published:** 2019-01-03

**Authors:** Eman Y. Gohar, Anthony K. Cook, David M. Pollock, Edward W. Inscho

**Affiliations:** Section of Cardio-Renal Physiology and Medicine, Division of Nephrology, Department of Medicine, University of Alabama at Birmingham, 720 20th St S, 35233, Birmingham, AL USA

**Keywords:** Endothelin, Kidney, Afferent arteriole, Sex

## Abstract

**Background:**

The pathogenesis of hypertension is distinct between men and women. Endothelin-1 (ET-1) is a potential contributor to sex differences in the pathophysiology of hypertension. ET-1 participates in blood pressure regulation through activation of endothelin A (ET_A_) and endothelin B (ET_B_) receptors including those in the vasculature. Previous studies demonstrated that sex and sex hormones evoke discrepancies in ET-1-mediated control of vascular tone in different vascular beds. However, little is known about sex- and sex hormone-related differences in ET-1-dependent renal microvascular reactivity. Accordingly, we hypothesized that loss of sex hormones impairs afferent arteriole reactivity to ET-1.

**Methods:**

Male and female Sprague Dawley rats were subjected to gonadectomy or sham surgery (*n* = 6/group). After 3 weeks, kidneys from those rats were prepared for assessment of renal microvascular responses to ET-1 (ET_A_ and ET_B_ agonist, 10^−12^ to 10^−8^ M) and sarafotoxin 6c (S6c, ET_B_ agonist, 10^−12^ to 10^−8^ M) using the blood-perfused juxtamedullary nephron preparation.

**Results:**

Control afferent arteriole diameters at 100 mmHg were similar between sham male and female rats averaging 14.6 ± 0.3 and 15.3 ± 0.3 μm, respectively. Gonadectomy had no significant effect on control arteriole diameter. In sham males, ET-1 produced significant concentration-dependent decreases in afferent arteriole diameter, with 10^−8^ M ET-1 decreasing diameter by 84 ± 1%. ET-1 induced similar concentration-dependent vasoconstrictor responses in sham female rats, with 10^−8^ M ET-1 decreasing the diameter by 82 ± 1%. The afferent arteriolar vasoconstrictor responses to ET-1 were unchanged by ovariectomy or orchiectomy. Selective ET_B_ receptor activation by S6c induced a concentration-dependent decline in afferent arteriole diameter, with 10^−8^ M S6c decreasing diameter by 77 ± 3 and 76 ± 3% in sham male and female rats, respectively. Notably, ovariectomy augmented the vasoconstrictor response to S6c (10^−12^ to 10^−9^ M), whereas orchiectomy had no significant impact on the responsiveness to ET_B_ receptor activation.

**Conclusion:**

These data demonstrate that sex does not significantly influence afferent arteriole reactivity to ET receptor activation. Gonadectomy potentiated the responsiveness of the afferent arteriole to ET_B_-induced vasoconstriction in females, but not males, suggesting that female sex hormones influence ET_B_-mediated vasoconstriction in the renal microcirculation.

## Background

The literature contains multiple studies showing that premenopausal women have lower blood pressure than age-matched men [1–3]. This male-female difference in blood pressure can be further exaggerated in different experimental models of hypertension [3], highlighting that mechanisms regulating blood pressure are sex-specific. The signaling pathways that control vascular tone are crucial for the maintenance of blood pressure [4]. Generally, vascular tone is under strict control relying on a balance between vasoconstrictor and vasodilator signaling mechanisms [4]. Alterations in these vasodilator and/or vasoconstrictor signals are implicated as central contributors to the pathogenesis of hypertension and even end organ injury.

Endothelin-1 (ET-1) contributes to regulation of vascular tone and has been implicated in the pathogenesis of different models of hypertension [5–8]. Since its discovery in 1988, ET-1 has emerged as one of the most potent pressor agents. Importantly, ET-1 contributes to basal vascular tone through activation of ET_A_ and ET_B_ receptors [9–11]. In the vasculature, ET-1 actions are primarily on the underlying vascular smooth muscle cells and endothelial cells, resulting in vasoconstriction or vasodilatation. Both ET_A_ and ET_B_ receptors are expressed on vascular smooth muscle cells, with ET_A_ receptors typically predominating. ET_B_ receptors are also present on endothelial cells where their activation results in vasodilatation via prostacyclin and nitric oxide release [9, 11]. Thus, the overall impact of ET-1 on vascular tone results from a balance between direct vasoconstrictor effects via ET_A_ and ET_B_ receptors on smooth muscle cells and vasodilator effects mediated by ET_B_ receptors on endothelial cells [12].

Vascular reactivity to ET-1 can be functionally distinct between sexes in different vascular beds. For example, aorta and mesenteric arteries from male, but not female, deoxycorticosterone acetate (DOCA)-salt-induced hypertensive rats display increased sensitivity to ET-1 and selective ET_B_ receptor activation [13, 14]. In vivo, mesenteric microvessels from female DOCA-salt-induced hypertensive rats were less responsive to ET_B_ receptor activation, compared to those from males [14].

Consistently, mesenteric arterioles from male spontaneously hypertensive rats are more sensitive to ET-1-induced vasoconstriction than those from females [15]. Alternatively, vasomotor reactivity of porcine skeletal muscle arteries to ET-1 is greater in females compared to males [16]. Female rats showed stronger intrahepatic vascular contractile responses to ET-1 than sham males [17]. Data from humans support the notion that vasoreactivity to ET-1 is sexually dimorphic. ET_B_ receptors in human cutaneous blood vessels mediate vasodilation in women [18], but they promote vasoconstriction in men [18]. Binding studies in human saphenous arteries show higher ET-1 binding to ET_A_ and ET_B_ receptors in men compared to women [19]. Similarly, human cerebral arteries from women are less responsive to ET-1 compared to men [20].

Collectively, ET-1 is a potent vasoactive agent and potential contributor to male-female differences in vascular tone and blood pressure control. In addition, data suggest important unrecognized roles for sex hormones in modulating vascular responsiveness to ET-1 [15, 17, 18, 20–22]. Afferent arterioles (AA) are the major resistance vessels in the kidney and key regulators of renal blood flow and glomerular filtration pressure. AA play a fundamental role in regulating renal hemodynamics and blood pressure [23–26]. Recently, model simulations suggest that female resistance to developing hypertension is related to sex differences in AA resistance [27]. However, little is known about the impact of sex and sex hormones on  ET-1 actions in the renal microcirculation. We have previously reported that renal medullary ET-1 reduces medullary blood flow in male, but not female, rats. This sex difference in ET-1-dependent effects on medullary blood flow was eliminated by orchiectomy and not ovariectomy [28]. Accordingly, in the current study, we hypothesized that loss of sex hormones impairs afferent arteriole reactivity to ET-1. Therefore, we determined the effects ET-1 (ET_A_ and ET_B_ receptor agonist) and S6c (selective ET_B_ receptor agonist) on AA diameter in kidneys from gonadectomized and gonadally intact male and female rats using the in vitro blood-perfused juxtamedullary nephron preparation.

## Methods

### Animals

Studies were performed on male and female age-matched (15–17 weeks old, *n* = 6/group) Sprague Dawley (SD) rats, purchased from Envigo laboratories (Indianapolis, IN). Body weight averaged 363 ± 7 and 250 ± 3 g for male and female rats, respectively. Rats were maintained on a normal salt diet (LABDIET NIH-31, Envigo, Indianapolis, IN) with free access to water. During the entire experimental period, animals were housed in a temperature-controlled room (~ 23 °C) with a 12:12-h light-dark cycle and a humidity of 55 ± 2%.

### Ovariectomy

Female rats (15–17 weeks old) were subjected to bilateral ovariectomy (OVX). Under 2% isoflurane anesthesia, small incisions were made on each side of the lower back to access the ovaries. Each ovary was isolated and surrounded by 2-0 silk thread then removed distal to the ligation. The skin incision was closed with wound staples. Sham operation involved exposure of the ovary without isolation. Buprenorphine (0.05 mg/kg, SC) was administered prior to and 24-h after OVX or sham operation for post-operative pain relief.

### Orchiectomy

Male rats (15–17 weeks old) were subjected to bilateral orchiectomy (ORX) or sham operation. Under isoflurane anesthesia (~ 2%), a small incision was made at the tip of the scrotum. The tunic was opened and the testes, cauda epididymis, vas deferens, and the spermatic blood vessels are exteriorized. The blood vessels and vas deferens were ligated with 2-0 silk thread. Then, the testes and epididymis were removed. The remaining tissue was returned into the sac, and the procedure was repeated for the other testes. The skin incision was closed with wound staples. Sham operation involved exposure of the testes without isolation. Buprenorphine (0.05 mg/kg, SC) was administered prior to and 24-h after ORX or sham operation for post-operative pain relief.

### Juxtamedullary nephron technique

Three weeks after gonadectomy or sham operation, videomicroscopy experiments were conducted in vitro using the blood-perfused juxtamedullary nephron technique as detailed previously [29, 30]. Briefly, two rats (kidney donor and an identically prepared blood donor) were anesthetized with pentobarbital sodium (50 mg/kg, IP) for each experiment. The right kidney from the kidney donor rat was cannulated via the superior mesenteric artery, harvested, and sectioned along the longitudinal axis on the dorsal two thirds of the kidney. Blood was collected from the kidney and blood donors into a heparinized syringe and processed collecting plasma and washing the erythrocyte fraction with saline. The plasma and washed erythrocyte were mixed to form a reconstituted blood perfusate with a hematocrit of ~ 33%. The harvested kidney was prepared for videomicroscopy. The perfusate was switched to the reconstituted blood. The focused image of the inner surface of the renal cortex was displayed on a video monitor and recorded on DVD for later analysis.

### Experimental protocol

The protocol began with a 5-min control period to establish control AA diameter with perfusion pressure held at 100 mmHg after an initial 15-min equilibration period. AA responses to ET receptor activation were determined by exposing the AA to increasing concentrations of ET-1 (ET_A_ and ET_B_ agonist, 10^−12^ to 10^−8^ M, American Peptide, Sunnyvale, CA) or sarafotoxin 6c (S6c, ET_B_ agonist, 10^−12^ to 10^−8^ M, American Peptide, Sunnyvale, CA) in the superfusate, and diameter was measured using an image shearing monitor. Steady-state AA diameter was calculated from the average of measurements made during the final 2 min of each treatment period.

## Results

### ET-1 effects on AA diameter in male and female rats

**Fig. 1:**
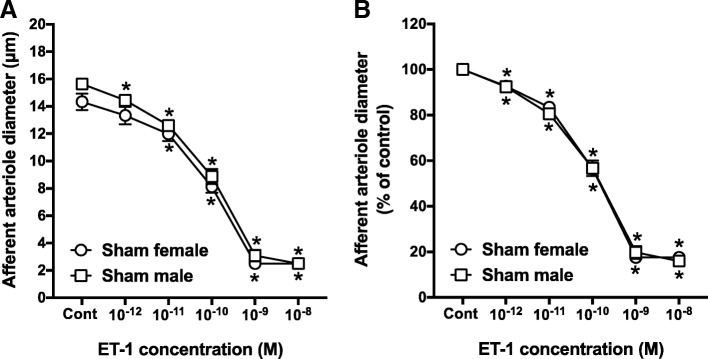
Afferent arteriole responses to ET-1 (ET_A_ and ET_B_ agonist, 10^−12^–10^−8^ M) in sham-operated male and female SD rats. **a** Data represent the actual diameters from each group. **b** The same data normalized as the percentage of control diameter for each group. Values are expressed as the mean ± SEM. *P < 0.05 vs. the control diameter in the same group (repeated measures two-way ANOVA, *n* = 6 in each group)

To study sex differences in ET receptor-induced vasoconstriction of preglomerular microvessels, we assessed the AA response to ET-1, a combined ET_A_ and ET_B_ agonist. ET-1 evoked concentration-dependent vasoconstriction of AA (Fig. 1). Control diameter averaged 15.6 ± 0.2 and 14.4 ± 0.6 μm for AA from male and female rats, respectively (Fig. 1a) and declined to 17.6 ± 0.8% and 16.0 ± 0.2% of the control diameter in response to increasing concentrations of ET-1 (Fig. 1b). No significant sex-related differences in the dose-response curves of ET-1 were observed.

### Effect of gonadectomy on AA responsiveness to ET-1

**Fig. 2:**
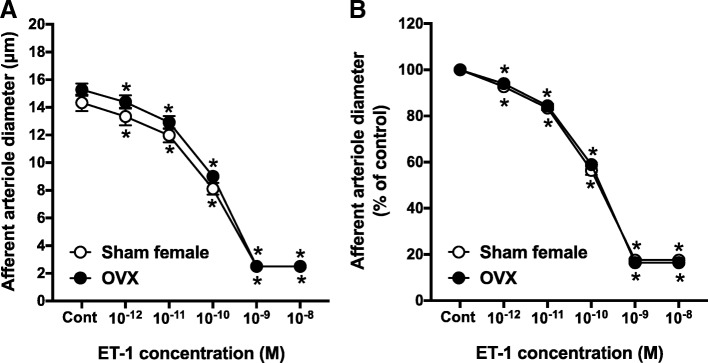
Afferent arteriole responses to ET-1 (ET_A_ and ET_B_ agonist, 10^−12^–10^−8^ M) in ovariectomized (OVX) and sham-operated female SD rats. **a** Data represent the actual diameters from each group. **b** The same data normalized as the percentage of control diameter for each group. Values are expressed as the mean ± SEM. *P < 0.05 vs. the control diameter in the same group (repeated measures two-way ANOVA, *n* = 6 in each group)

To assess the role of male and female sex hormones on ET-1-induced vasoconstriction of AA, we determined the effect of OVX and ORX on AA responsiveness to ET-1 compared to vessels from sham-operated male and female rats, respectively. Control diameter in AA from OVX rats averaged 14.4 ± 0.5 μm and declined to 16.4 ± 0.5% of the control diameter in response to 10^−8^ M ET-1 (Fig. 2a). AA reactivity to ET-1 in arterioles from OVX rats was similar to responses from sham-operated females (Fig. 2b).

**Fig. 3:**
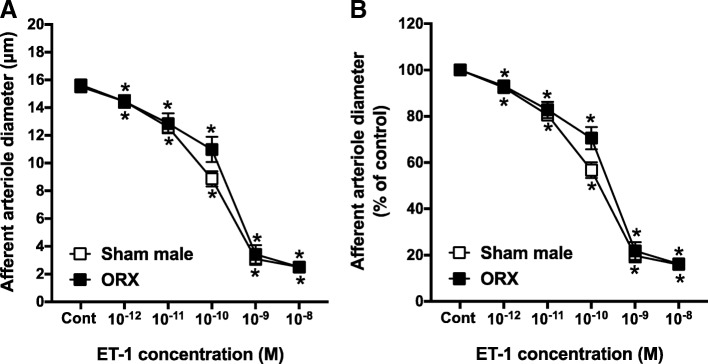
Afferent arteriole responses to ET-1 (ET_A_ and ET_B_ agonist, 10^−12^–10^−8^ M) in orchiectomized (ORX) and sham-operated male SD rats. **a** Data represent the actual diameters from each group. **b** The same data normalized as the percentage of control diameter for each group. Values are expressed as the mean ± SEM. *P < 0.05 vs. the control diameter in the same group (repeated measures two-way ANOVA, *n* = 6 in each group)

Control diameter in AA from ORX rats averaged 15.3 ± 0.5 μm, which was not significantly different from the diameter of arterioles from sham-operated male rats (Fig. 3a). In response to increasing concentrations of ET-1, AA diameter declined to 16.2 ± 0.3% of the control diameter (Fig. 3b).

### Effects of S6c on AA reactivity in male and female rats

**Fig. 4:**
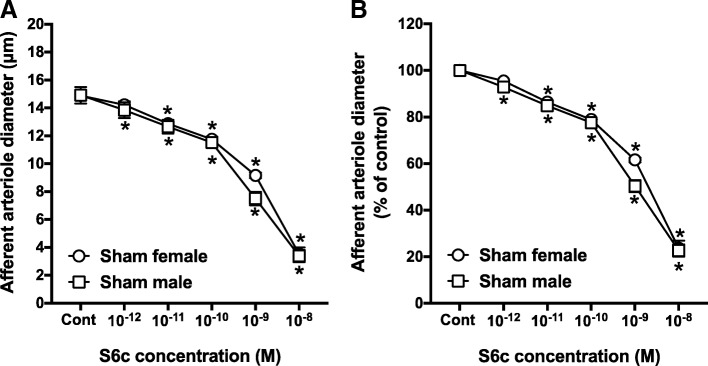
Afferent arteriole responses to S6c (ET_B_ agonist, 10^−12^–10^−8^ M) in sham-operated male and female SD rats. **a** Data represent the actual diameters from each group. **b** The same data normalized as the percentage of control diameter for each group. Values are expressed as the mean ± SEM. *P < 0.05 vs. the control diameter in the same group (repeated measures two-way ANOVA, *n* = 6 in each group)

To determine if there are any male-female differences in ET_B_-mediated control of renovascular tone, we determined the effect of the selective ET_B_ receptor agonist, S6c, on AA diameter. Control arteriole diameter averaged 14.9 ± 0.6 and 14.9 ± 0.3 μm in vessels from male and female rats, respectively (Fig. 4a). S6c decreased the arteriole diameter to 23.7 ± 3.3% of the control diameter in arterioles from female rats, compared with 22.7 ± 2.6% in arterioles from male rats (Fig. 4b). Thus, AA reactivity to S6c was similar in these male and female rats.

### Effect of gonadectomy on AA responsiveness to ET_B_ receptor activation

**Fig. 5:**
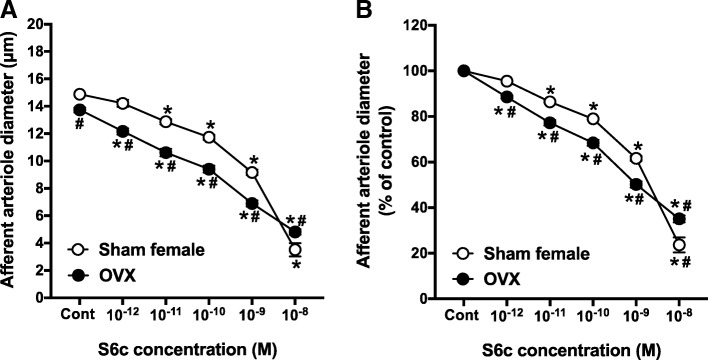
Afferent arteriole responses to S6c (ET_B_ agonist, 10^−12^–10^−8^ M) in ovariectomized (OVX) and sham-operated female SD rats. **a** Data represent the actual diameters from each group. **b** The same data normalized as the percentage of control diameter for each group. Values are expressed as the mean ± SEM. *P < 0.05 vs. the control diameter in the same group. ^#^*P* < 0.05 vs. the corresponding point in sham-operated females (repeated measures two-way ANOVA, *n* = 6 in each group)

**Fig. 6:**
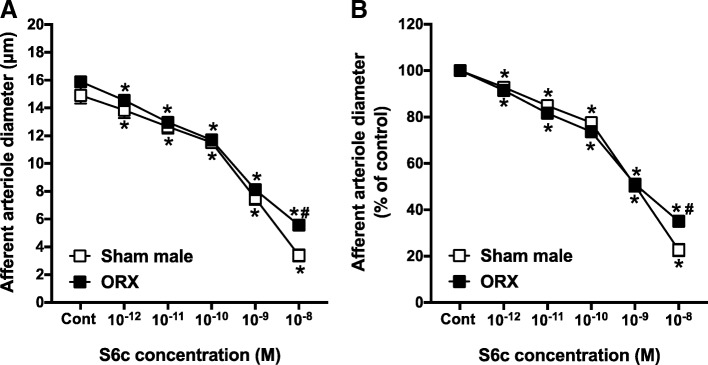
Afferent arteriole responses to S6c (ET_B_ agonist, 10^−12^–10^−8^ M) in orchiectomized (ORX) and sham-operated male SD rats. **a** Data represent the actual diameters from each group. **b** The same data normalized as the percentage of control diameter for each group. Values are expressed as the mean ± SEM. *P < 0.05 vs. the control diameter in the same group. ^#^*P* < 0.05 vs. the corresponding point in sham-operated males (repeated measures two-way ANOVA, *n* = 6 in each group)

The S6c concentration-response curve in female rats was shifted to the left by OVX. In kidneys from OVX rats, increasing concentrations of S6c decreased arteriole diameter to 88.6 ± 0.7%, 77.3 ± 1.2%, 68.6 ± 1.3%, 50.2 ± 1.3%, and 35.1 ± 1.5% of control diameter. The magnitudes of these vasoconstrictions were significantly greater than the 95.5 ± 0.4%, 86.5 ± 0.3%, 79.0 ± 0.5%, 61.6 ± 2%, and 23.7 ± 3.3% changes in diameter observed from sham female rats (Fig. 5b). Thus, OVX significantly potentiated the vasoconstrictor responses to S6c (10^−12^–10^−9^ M), compared to sham females. Alternatively, the S6c concentration-response curve in arterioles from ORX rats was not different from responses observed from arterioles in sham-operated male kidneys (Fig. 6). Accordingly, AA reactivity to S6c was significantly potentiated by OVX, whereas ET_B_-induced AA vasoconstriction was not significantly changed by ORX (Figs. 5 and 6).

## Discussion

The present study represents an initial determination of afferent arteriole vasoreactivity to ET-1 and S6c in gonadectomized and gonadally intact male and female rats. The main finding of the current study is that OVX, a model of surgical menopause, augmented the vasoconstrictor response to ET_B_ receptor activation, reinforcing the suggestion that ET_B_ receptor-mediated control of the AA resistance is regulated by ovarian hormonal status. This mechanism may contribute to increased incidence of cardiovascular and renal diseases in postmenopausal women.

As a potent endothelium-derived vasoactive agent, ET-1 plays a fundamental role in regulating vascular function and blood pressure in a sex-specific manner. Administration of bosentan, a mixed ET_A_ and ET_B_ antagonist, reduces blood pressure more effectively in male, compared to female, DOCA-salt-induced hypertensive rats [14], whereas ET_A_ receptor antagonism by the selective ET_A_ receptor antagonist, ABT-627, reduced blood pressure to a greater extent in female than male DOCA-salt-induced hypertensive rats [22]. While the DOCA-salt model is largely neurogenic, it also involves multiple organ systems. Cardiovascular and renal components are considered important players in the pathogenesis of hypertension in this model [31]. Renal autoregulation is lost in this DOCA model of hypertension [32]. Responsiveness of the afferent arteriole to purinergic (P2X_1_) receptor activation is impaired in this hypertensive model [32] establishing a link between responsiveness of the renal microvasculature and DOCA-salt hypertension.

In the present study, combined ET_A_ and ET_B_ receptor activation with ET-1, or selective ET_B_ receptor activation with S6c, evoked concentration-dependent vasoconstriction of AA from male rats. This agrees with previous findings demonstrating that both ET_A_ and ET_B_ receptors participate in ET-1-mediated vasoconstriction of AA in male rats [33]. Our current data show similar/comparable vasoconstrictor responses to ET-1 and S6c in AA from female rats compared to males. Similarly, no differences were observed between men and women in the vasoconstrictor effect of ET-1 on forearm blood flow [34]. Unfortunately, there are no ET_A_-specific agonists and so the role of the ET_A_ receptor can only be inferred from the difference between responses obtained with combined ET_A_ and ET_B_ agonists versus selective ET_B_ agonists.

The current study adds to accumulating evidence implicating an essential role for sex hormones in differential responses evoked by ET receptor antagonists in females. OVX augments the antihypertensive effect of bosentan [35]. Data suggest that ET receptor antagonists have estrogen-like vasoprotective effects in OVX rats [36], indicating that ET receptor antagonists may be a useful therapeutic option for preventing vascular disease in postmenopausal women [36, 37]. The current study demonstrates that OVX potentiates AA vasoconstrictor responses to selective ET_B_ receptor activation by S6c, but not to combined ET_A_ and ET_B_ receptor activation with ET-1. This finding provides evidence that ovarian hormones influence ET_B_ receptor function in the renal microcirculation. Whether this is due to reduced endothelial ET_B_ receptors or increased vascular ET_B_ receptors remains to be determined. Interestingly, our lab recently reported that OVX decreased total ET_B_ receptor mRNA expression in the renal cortex and increased ET_B_ receptor expression in the renal medulla [38], but it remains unclear whether this represents a functional change in vascular versus tubular ET_B_ expression.

Clinical and animal studies pointed to a central role for female sex hormones, particularly estradiol (E_2_), in regulating vascular tone. E_2_ supplementation to postmenopausal women improves endothelium-dependent flow-mediated vasodilation in brachial artery [39]. Treatment of human coronary endothelial cells with E_2_ increases basal and ATP-induced NO release [40]. In addition, E_2_ reduces mouse cerebral artery tone through endothelial NO synthase and cyclooxygenase-dependent mechanisms [41]. Nicotine reduced renal perfusion pressure in isolated perfused kidneys from proestrus female rats via a NOS-dependent pathway [42]. This vasodilatory effect of nicotine was attenuated by OVX and restored by supplementation of OVX rats with E_2_, suggesting a faciliatory effect for E_2_ on NOS signaling in the renal vasculature [42]. Similarly, renovascular responses to β-adrenoceptor activation by isoprenaline in female rats were reduced by OVX and restored by supplementation of OVX rats with E_2_ [43]. Adenosine receptor-mediated renal vasodilations induced by 5′-N-ethylcarboxamidoadenosine (NECA) in perfused kidneys were reduced in OVX rats and restored after treatment with E_2_ or progesterone [44]. Further studies are needed to identify the individual and combined effects of E_2_ and progesterone on the responsiveness of the renal microvasculature to ET-1 receptor activation.

Previous data also suggest a link between the male sex hormones, specifically testosterone, and vascular tone in different vascular beds. Testosterone treatment increased ET-1 constriction of porcine coronary artery rings [45]. ORX increased expression of ET_A_ and ET_B_ receptors in the rat portal veins, and this increase was completely reversed by testosterone replacement [46]. Human data suggest that androgen suppression improves skin microcirculatory vasodilator responsiveness to local heating in polycystic ovary syndrome via ET_A_ and ET_B_ receptors [47]. In contrast, the present study did not show a significant effect of ORX on AA responsiveness to combined ET_A_ and ET_B_ or selective ET_B_ receptor activation, suggesting that testosterone depletion may not have a direct effect on ET receptor function in the AA. Interestingly, our lab previously reported that renal medullary ET-1 reduces medullary blood flow in male, but not female, rats. ORX eliminated ET-1-dependent decreases in medullary blood flow, but OVX had no detectable effect on this sex difference [28].

Receptor expression measurements have previously shown that the distribution of ET_A_ and ET_B_ receptor subtypes in different vascular tissues appears to be sex- and sex hormone-dependent [12]. The expression of ET_A_ and ET_B_ receptors, as well as their ratio, is higher in the saphenous veins from men, compared to those from women [19]. In contrast, the expression of ET_B_ receptors is higher in human cerebral arteries from women than those from men [20]. ET_B_ receptor expression is higher in mesenteric arteries from OVX DOCA-salt-induced hypertensive rats, compared to intact females [35]. In renal cortex, both ET_A_ and ET_B_ receptor expressions were significantly attenuated by OVX, and this reduction was not evident in OVX rats supplemented with E_2_ [38].

In addition to sex differences in receptor expression, intracellular signaling pathways utilized by ET receptors may be other potential contributors to sex differences in response to ET receptor activation. Giachini et al. revealed that the extracellular signal-regulated kinase (ERK)1/2 pathway activated by ET-1 contributes to the augmented vasoconstriction in aorta and mesenteric arteries in male, compared to female DOCA-salt-induced hypertensive rats [48]. Further mechanistic studies are needed to compare downstream signaling pathways activated by ET_A_ and ET_B_ receptors in vascular smooth muscle and endothelial cells.

To our knowledge, this is the first direct comparison of AA responsiveness to ET-1 receptor activation in male and female rats with and without gonads. Our data show no significant sex hormone-related differences in the vasoconstrictor response to combined ET_A_ and ET_B_ receptor activation. However, gonadectomy enhanced AA sensitivity to ET_B_-induced vasoconstriction in females, but not males. These data suggest that ovarian hormones can influence microvascular reactivity to ET_B_ receptor activation in the renal microcirculation.

### Study limitations

Ovariectomy, a model of surgical menopause, was used to deplete ovarian hormones in the current study. Potential extrapolation of the current findings to postmenopausal conditions needs to be verified as vascular aging might have an impact on responsiveness to vasoactive agents beside the impact of ovarian hormonal depletion.

In the current study, the time elapsed between gonadectomy and assessment of renovascular function was 3 weeks. We have previously published evidence in rats with the same background that this time interval is sufficient to significantly drop their serum E_2_ level [38]. It is feasible that the changes in responsiveness to ET-1 may be more profound after more prolonged deprivation of the sex hormones. Future studies are needed to address the impact of the timing hypothesis on the renovascular responsiveness to different vasoactive agents including ET-1.

## Conclusion

This study demonstrates an important modulatory role of ovarian hormones on ET_B_ receptor function in AA that may have a considerable impact on cardiovascular and renal health in the hormonally deficient female population. Future studies are needed to clarify the effects of the ovarian hormones on vascular smooth muscle versus endothelial ET receptor expression, signaling, and function.
